# LIFETIME PATTERNS OF EXPOSURE TO JOB COMPLEXITY AND LATER LIFE COGNITION

**DOI:** 10.1093/geroni/igae098.2375

**Published:** 2024-12-31

**Authors:** Qize Chen, Joanne Hsu, Yun taek Oh, Jacqui Smith, Amanda Sonnega

**Affiliations:** University of Michigan, Ann Arbor, Michigan, United States; University of Michigan, Ann Arbor, Michigan, United States; University of Nevada Reno, Reno, Nevada, United States; University of Michigan, Ann Arbor, Michigan, United States; University of Michigan, University of Michigan, Michigan, United States

## Abstract

A growing body of evidence establishes a positive connection between cognitively engaging activities and later life cognitive functioning. Cognitively stimulating work—jobs that involve complex tasks and demands—is gaining increased research attention in this regard. Given data limitations to date, however, less research has focused on exposure to job complexity over the entire adult life course. We leverage new retrospective data from the Health and Retirement Study on job histories spanning early adulthood through midlife in combination with the rich prospective survey measurement of cognitive functioning through mid- and later-life to study this association (N=6,744; 41.24% men). We link early life job histories to historical information from the Occupational Information Network to characterize lifetime job complexity exposure. We find a lifetime pattern of annual exposure to job complexity (those demanding complex problem solving, critical thinking, inductive reasoning, guiding and directing others, making decisions, and coordinating/leading others) of a rapid increase in the 20s, gradually increasing through the 30s, 40s, and 50, and decreasing after age 60. We find no significant cohort differences (people born 1924-1941 vs after), but there are gender and racial/ethnic differences. Women’s annual exposure to job complexity tends to start at a higher level compared to that of men, but by their early 30s, men’s exposure exceeds women’s and remains higher. Non-Hispanic whites have significantly higher lifetime job complexity exposure relative to Non-Hispanic Black and Hispanic individuals. These patterns shed light on life course work exposures and demographic factors associated with differences in later-life cognitive functioning.

